
JOURNAL INFORMATION
==============================
NLM Title Abbreviation: Wirtschaftsdienst
Journal ID: Wirtschaftsdienst
NLM Journal ID: 101086612

Wirtschaftsdienst (Hamburg, Germany : 1949)

ISSN: 0043-6275

ARTICLE INFORMATION
==============================
PMCID: PMC7174821
PMID: 32336797
DOI: 10.1007/s10273-020-2621-7
Article ID: 2621
Article version: 1
Subjects: Kurz Kommentiert

Sicherheit in unsicheren Zeiten

Corona crisis: Security in uncertain times

Dudenhöffer Ferdinand ferdinandwalter.dudenhoeffer@unisg.ch

grid.15775.31 0000 0001 2156 6618 Institut für Customer Insight (ICI-HSG), Universität St. Gallen, Bahnhofstrasse 8, 9000 St. Gallen, Switzerland
Electronic publication date: 2020 Apr 22
Print publication date: 2020
Volume: 100
Issue: 4
First page: 231
Last page: 231
Copyright: © Der/die Autor(en) 2020
License: Open Access Dieser Artikel wird unter der Creative Commons Namensnennung 4.0 International Lizenz veröffentlicht, welche die Nutzung, Vervielfältigung, Bearbeitung, Verbreitung und Wiedergabe in jeglichem Medium und Format erlaubt, sofern Sie den/die ursprünglichen Autor(en) und die Quelle ordnungsgemäß nennen, einen Link zur Creative Commons Lizenz beifügen und angeben, ob Änderungen vorgenommen wurden.
Die in diesem Artikel enthaltenen Bilder und sonstiges Drittmaterial unterliegen ebenfalls der genannten Creative Commons Lizenz, sofern sich aus der Abbildungslegende nichts anderes ergibt. Sofern das betreffende Material nicht unter der genannten Creative Commons Lizenz steht und die betreffende Handlung nicht nach gesetzlichen Vorschriften erlaubt ist, ist für die oben aufgeführten Weiterverwendungen des Materials die Einwilligung des jeweiligen Rechteinhabers einzuholen.
Weitere Details zur Lizenz entnehmen Sie bitte der Lizenzinformation auf http://creativecommons.org/licenses/by/4.0/deed.de.
License URL: https://creativecommons.org/licenses/by/4.0/

issue-copyright-statement: © The Author(s) 2020

==============================
DOI: 10.1007/s10273-020-2621-7

Corona-Krise

### Sicherheit in unsicheren Zeiten

Das Coronavirus stellt die Weltwirtschaft vor eine nie dagewesene Herausforderung. Mit Shutdowns soll die Verbreitung des Virus gestoppt werden, damit die Gesundheitssysteme nicht ins Chaos laufen. Wie groß die weltweite Rezession sein wird, kann niemand sagen. Sicher ist nur, der Einbruch des globalen Bruttoinlandsprodukts (BIP) wird größer sein als zu Zeiten der Finanzkrise. Billionen an Liquiditätshilfen werden in die Wirtschaft gepumpt, um eine nicht mehr beherrschbare Insolvenzwelle zu vermeiden. Billionen werden gebraucht werden, um nach den Shutdowns die Nachfrage wieder zu beleben. Der private Konsum spielt dabei die Schlüsselrolle. Investitionen werden auch bei noch so lukrativen, negativen Zinssätzen nicht in Gang kommen. Wer investiert schon, wenn er keine Nachfrage sieht? Und beim privaten Konsum sind es nicht die Produkte des täglichen Bedarfs, sondern höherwertige Konsumprodukte, wie etwa Autos, Wohnungseinrichtungen oder Weltreisen, die Wirtschaftswachstum erzeugen.

2019 wurden weltweit 80 Mio. Pkw-Neuwagen verkauft. Bei einem Durchschnittspreis von 25.000 Euro entspricht dies 2 Billionen Euro Umsatz. Dies zeigt, wie wichtig der weltweite Automarkt für die Wiederbelebung der Wirtschaft nach den Shutdowns ist. Monetaristen würden Hubschrauber über die Lande fliegen lassen und Geldscheine abwerfen. Mit Recht hat Keynes gesagt, die Konsumenten würden die Geldscheine vergraben. Wer Angst hat, seinen Job zu verlieren, kauft keine hochwertigen Konsumgüter. Der größte Kundenwert in unsicheren Zeiten ist Sicherheit. Helikoptergeld wäre sinnvoll, wenn man damit die Unsicherheit nehmen könnte.

2009 war der US-Automarkt nach der Lehman-Pleite mit dem größten Einbruch konfrontiert. Wurden 2007 noch 16,2 Mio. Neuwagen in den USA verkauft, waren es 2009 nur noch 10,4 Mio. Der fast „ewige“ Weltmarktführer General Motors überlebte den Absturz nicht und musste in der Insolvenz mit Staatsfinanzen aufgefangen werden. Nicht viel besser ging es Chrysler. Hyundai verkaufte 2007 in den USA 467.000 Fahrzeuge und hatte einen Marktanteil von 2,9 %. 2009 sank der Hyundai-Verkauf nur um 6,8 %. In der größten Krise steigerte Hyundai seinen Marktanteil aber auf 4,2 %. Was hatte Hyundai anders gemacht? Hyundai startet eine große Werbekampagne mit dem Titel „Certainty in Uncertain Times“. Einige Jahre zuvor hatte Hyundai die Garantie auf seine Autos erheblich ausgeweitet, also das Produktrisiko dem Kunden abgenommen. „Today we‘re introducing Hyundai Assurance. Right now, buy any new Hyundai, and if in the next year you lose your income, we‘ll let you return it.“ In der großen Krise hatte Hyundai die Kunden vom wirtschaftlichen Risiko freigestellt, deshalb war Hyundai erfolgreich.

Heute sind mit sogenannten Car-Abos oder Car-Subscriptions von Start-Up-Unternehmen wie Like2Drive, Cluno und dem Vermieter Sixt Produkte im Markt, die ähnliche Wirkung entfalten. Die Finanzierer der klassischen Autobauer laufen der Zeit hinterher und bieten so gut wie keine dieser Produkte an. Car-Abo bedeutet, dass man für einen fixen Monatsbetrag einen Neuwagen für sechs, zwölf, 18 oder 24 Monate nutzen kann. Dabei sind außer Kraftstoffkosten alle Kosten bereits mit der Monatsrate abgegolten – berechenbare Mobilität, wenn Helikoptergeld kommt. Zusätzlich lässt sich die Berechenbarkeit erweitern, wenn man die vorzeitige Rückgabe auch bei einem Zwölf- oder 24-Monatsvertrag ermöglicht. Die Autobauer hätten die Chance, dem Kunden das wirtschaftliche Risiko abzunehmen.

Mit Car-Abos und der Hyundai-Idee könnten sich die Autobauer zum Teil selbst aus dem Sumpf ziehen. Die Größe der Verwerfung durch die Shutdowns macht allerdings zusätzliche Hilfen notwendig. Helikoptergeld kann man den Autokäufern in Form von Rabatten geben. Sehr einfach, unkompliziert und schnell wirksam wäre die Aussetzung der Mehrwertsteuer und das am besten EU-weit. Der Durchschnittsneuwagen in Deutschland kostet heute 35.000 Euro. Ohne Mehrwertsteuer könnte das Fahrzeug für 29.412 Euro erstanden werden. Ein „Staatsrabatt“ oder Helikoptergeld von 5.588 Euro, das man nicht vergraben kann. Dies kombiniert mit der Absicherung des wirtschaftlichen Käuferrisikos schafft Kundenwert in schwierigen Zeiten.

Natürlich ist das Modell nicht auf Autos beschränkt. Es lässt sich auf alle hochwertigen Konsumprodukte einfach, ohne Formulare anwenden. Man muss nur den unteren Preispunkt, also etwa 10.000 Euro und die Dauer der Aktion festlegen. Sofort sehen die Kunden die Preisvorteile, und die Unternehmen könnten das Hyundai-Modell übernehmen. All diejenigen, die das Risiko lieber beim Kunden lassen, werden durch einen Marktanteilsverlust bestraft. Die Hyundais und unsere Gesellschaft werden zu Siegern, denn der Einbruch beim BIP kann gezielt durch private Nachfrage ausgeglichen werden. Staatliche Nachfrage und noch höhere Schuldenlasten wären mehr als kontraproduktiv.

Ferdinand Dudenhöffer
Universität St. Gallen
ferdinandwalter.dudenhoeffer@unisg.ch

© Der/die Autor(en) 2020. Open Access: Dieser Artikel wird unter der Creative Commons Namensnennung 4.0 International Lizenz (https://creativecommons.org/licenses/by/4.0/deed.de) veröffentlicht.

Open Access wird durch die ZBW – Leibniz-Informationszentrum Wirtschaft gefördert.
